# Integrated multi-omics reveals the impact of ruminal keystone bacteria and microbial metabolites on average daily gain in Xuzhou cattle

**DOI:** 10.1128/spectrum.00769-25

**Published:** 2025-06-30

**Authors:** Yingying Jiang, Zhenjiang An, Wenjie Li, Shuwen Xia, Qiang Ding, Jifeng Zhong, Huili Wang, Yan Xu, Kunlin Chen, Yangyang Shen

**Affiliations:** 1 Institute of Animal Science, Jiangsu Academy of Agricultural Sciences 117941, Nanjing, Jiangsu, China; 2 College of Animal Science and Technology, Nanjing Agricultural University 70578, Nanjing, Jiangsu, China; 3 Jiangsu Provincial Engineering Research Center of Precision Animal Breeding, Nanjing, Jiangsu, China; 4 Jiangsu Provincial Station of Animal Husbandry, Nanjing, Jiangsu, China; Chengdu University, Chengdu, Sichuan, China

**Keywords:** Xuzhou cattle, daily weight gain, rumen microbiome, metabolites, machine learning

## Abstract

The rumen microbiome plays a crucial role in determining the metabolic and digestive efficiency of livestock. Despite its crucial role, the impact of the rumen microbiome on average daily gain (ADG) in Xuzhou cattle remains underexplored. Xuzhou cattle is a well-known breed in China, renowned for rapid growth and superior meat quality. We selected 10 individuals from the Xuzhou cattle population and categorized Xuzhou cattle into High-ADG and Low-ADG groups and analyzed their rumen microbiota. Through comprehensive metagenomic and metabolomic analyses, we characterized the microbial diversity and functional composition of the rumen microbiome, uncovering distinct taxonomic and functional alterations associated with ADG. Thirteen kingdoms, 224 phyla, and over 16,000 species were identified, and principal coordinates analysis (PCoA) indicated significant microbial differentiation between the two groups on phylum, genus, and species levels (*P* < 0.05). Notably, Lentisphaerae, along with several other genera and species, presented a higher abundance in High, suggesting a potential connection with enhanced growth performance. Further functional annotation revealed that the High group displayed enriched carbohydrate and amino acid metabolism pathways, with a greater abundance of carbohydrate-active enzymes (CAZymes), particularly those involved in the degradation of complex carbohydrates. The Low-ADG group exhibited reduced metabolic activity in these pathways. Metabolomic analysis revealed 10 significantly altered metabolites, including gamma-glutamyltyrosine and N-acetylaspartic acid, which were upregulated in the High-ADG group, indicating their potential role in growth promotion. Spearman’s rank correlation analysis further uncovered significant interactions between key microbiomes and metabolites, which correlated with ADG. Random forest analysis identified Victivallales and Lentisphaerae as key taxa, with gamma-glutamyltyrosine and Asp-Phe emerging as predictive biomarkers for ADG.

**IMPORTANCE:**

This study identifies key microbiota (Victivallales and Lentisphaerae) and metabolites (gamma-glutamyltyrosine, Asp-Phe, N-acetylaspartic acid, Gly-Phe) that positively regulate average daily gain (ADG) in Xuzhou cattle through amino acid metabolism. This fundamental information is vital for the development of potential manipulation strategies to improve the daily gain level through precision feeding.

## INTRODUCTION

The growth traits of beef cattle are fundamental indicators used to assess not only their production performance but also their overall economic value. These traits span key stages of development, starting from birth and continuing through to maturity. The weight at 1 year and, later, at maturity are important indicators of development, directly influencing the slaughter weight and the yield of high-quality meat. Furthermore, average daily gain (ADG) serves as one of the most influential metrics for evaluating growth performance. It quantifies the rate at which an animal accumulates weight over time and is intrinsically tied to feed conversion efficiency, which dictates production costs and time to market. Consequently, optimizing ADG is central to improving overall farm profitability. The rate of growth in beef cattle is influenced by a wide array of factors, including management practices (1), genetics (2), and nutrition (3). Management factors such as feed quality, health protocols, and environmental conditions have long been recognized for their direct impact on cattle growth and meat quality. Genetics, through the inheritance of specific traits, also plays a vital role in determining an animal’s growth rate and meat production potential. However, one of the most critical, yet often overlooked, aspects that significantly affects beef cattle growth is the rumen microbiota (4, 5). As a fundamental component of the cattle’s digestive system, the rumen microbiota, which consists of a complex array of bacteria, protozoa, fungi, and archaea, plays a vital role in breaking down fibrous plant material and converting it into digestible nutrients. Approximately 70% of a beef cattle’s energy requirements are met through the volatile fatty acids (VFAs) produced by rumen microbial fermentation (6), with a significant portion of the protein demand being fulfilled by microbial protein synthesis (7). This microbial process is vital not only for digestion but also for optimizing the conversion of nutrients from feed into body mass. In addition to its role in energy metabolism, the rumen microbiota also produces numerous secondary metabolites that act as regulatory signals within the gut-brain (8) and gut-liver axes (9–11), influencing various physiological processes, including body composition (12), bone density (13), muscle development (14), and even feeding behavior (15). These complex interactions highlight the central role of the microbiota in regulating the growth efficiency of beef cattle.

Rumen microbial metabolism is critical for beef cattle growth and quality (16). Microbial communities in the rumen ferment fibrous plant material and synthesize key nutrients, including VFAs and microbial protein, which are vital for energy production and muscle development (17). VFAs, the primary byproducts of rumen fermentation, provide up to 70% of a beef cattle’s energy, while microbial protein satisfies a substantial portion of its amino acid needs. Recent studies have identified key microbial species involved in carbohydrate degradation for precision. For instance, Bacteroidetes and Firmicutes dominate polysaccharide degradation (18), producing VFAs for energy, while Selenomonas ruminantium aids in breaking down fibrous materials (19). Microbial diversity supports rumen function and enhances growth performance. Advances in molecular biology, especially next-generation sequencing and metagenomics, have greatly enhanced our understanding of the rumen microbiome’s role in cattle growth (20). Metagenomics enables a comprehensive, culture-independent analysis of microbial communities, revealing genetic diversity and functional potential. Metagenomics enables a comprehensive, 80 culture-independent analysis of microbial communities (21, 22). Recent findings show that Bacteroidetes and Firmicutes are key in polysaccharide breakdown, while phyla like Verrucomicrobia and Spirochaetes contain enzymes that degrade complex carbohydrates. These insights underline the complexity of microbial interactions and their impact on feed utilization and energy metabolism (23). Metagenomics has also uncovered microbial species involved in producing metabolites that influence beef quality, such as those responsible for meat flavor (24–27), highlighting the power of multi-omics approaches in understanding the biochemical pathways contributing to meat quality. The analysis of complex traits benefits significantly from the application of multi-omics strategies (28–31).

Xuzhou cattle, also known as Xuzhou Yellow Cattle, are renowned for their exceptional meat quality and flavor, particularly in China, where they are commonly referred to as “Xuzhou Big Yellow Cattle.” Over recent years, as social and economic conditions have evolved, Xuzhou cattle have transitioned from primarily being draught animals to a more prominent role in meat production, owing to their rapid growth, superior meat quality, and ease of fattening. Despite their economic value, research on Xuzhou cattle growth and fattening, especially ADG-related mechanisms, is limited. While there has been some research on the genetic and nutritional aspects of beef cattle growth, the rumen microbiome’s role in regulating growth and quality in Xuzhou cattle has not been thoroughly explored. This study aims to address this gap by investigating the relationship between rumen microbial composition and metabolic profiles in two groups of Xuzhou cattle with different average daily gain phenotypes. By identifying microbial markers associated with growth performance, this research seeks to provide valuable insights into how management and nutritional strategies can be optimized to enhance growth efficiency and beef quality in Xuzhou cattle.

## RESULTS

### Profiling of the rumen metagenome

**Fig 1:**
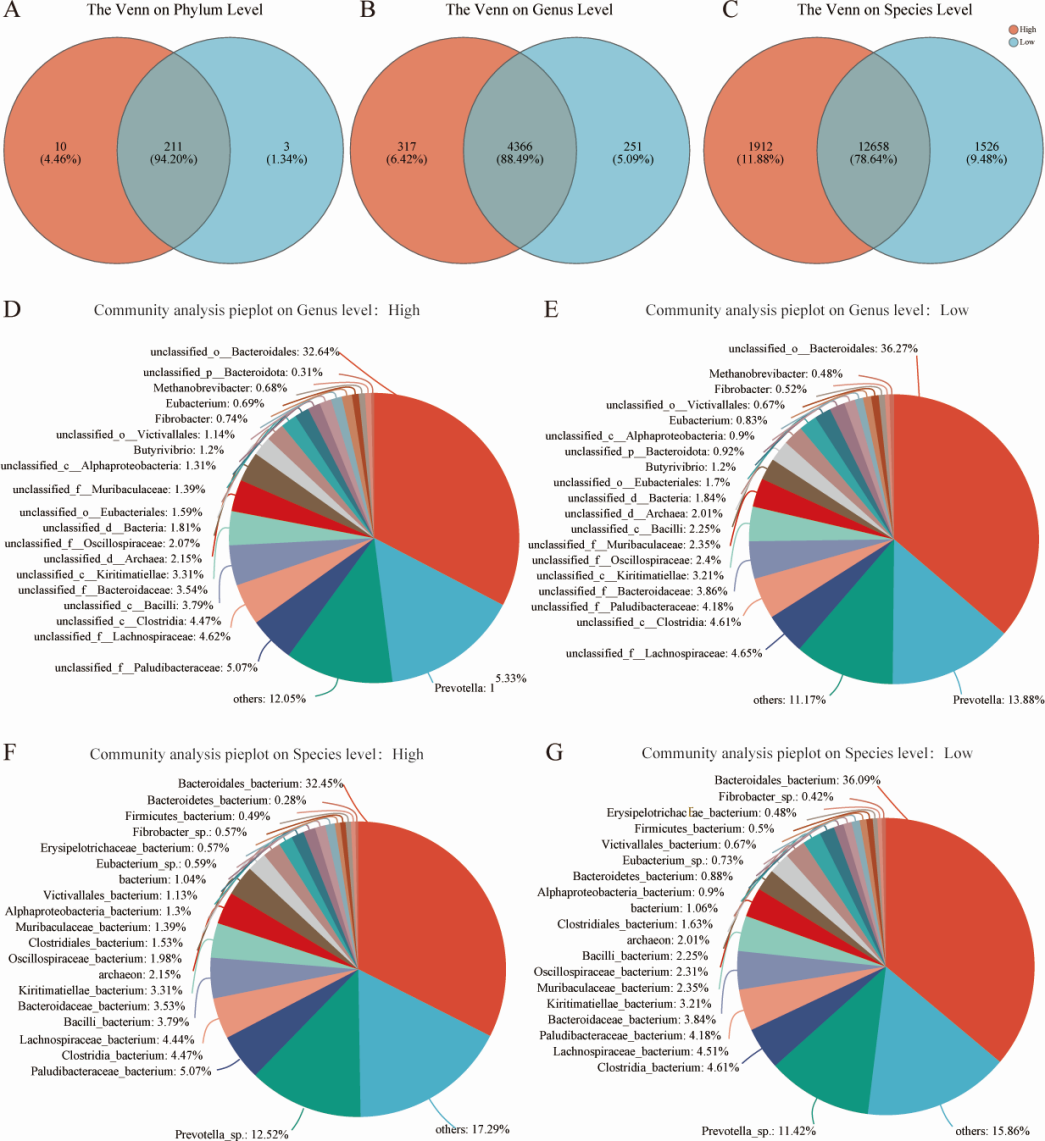
Comparative analysis of rumen microbial communities between High and Low groups in Xuzhou cattle. Microbial community structure in the rumen of Xuzhou cattle on phylum level (**A**), genus level (**B**), and species level (**C**). Community analysis pie plot of High group (**D**) and Low group (**E**) on genus level. Community analysis pie plot of High group (**F**) and Low group (**G**) on species level**.**

Metagenomic sequencing yielded a total of 452,163,864 reads, with an average of 45,216,386.4 ± 1,413,603.36 reads (mean ± standard error of the mean) per sample. Following quality control and the removal of host genes, 449,114,718 reads were retained, with an average of 44,911,471.8 ± 1,417,191.28 reads per sample. After further quality filtering and host gene removal, 366,753,826 reads remained, with an average of 36,675,382.6 ± 1,117,451.34 reads per sample (Table S1). A comprehensive taxonomic analysis identified 13 kingdoms, 224 phyla, 458 classes, 977 orders, 2,018 families, 4,934 genera, and 16,096 species (Tables S2 and S8). At the phylum level (Fig. 1A), the High group and Low group shared 211 species (94.20%), with 10 species (4.46%) specific to the High group and three species (1.34%) specific to the Low group. At the genus level (Fig. 1B), 4,366 species (88.49%) were common between both groups, while 317 species (6.42%) were unique to the High group and 251 species (5.09%) to the Low group. At the species level (Fig. 1C), 12,658 species (78.64%) were shared, with 1,912 species (11.88%) unique to the High group and 1,526 species (9.48%) unique to the Low group (Fig. S1). Principal coordinates analysis (PCoA) revealed that the first and second principal components accounted for 51.22% and 26.24%, respectively, of the total variance at the phylum level. At the genus level, the first and second components explained 47.31% and 18.15%, respectively, while at the species level, they accounted for 45.29% and 17.96% (Fig. S1).

### Compositional profiles and taxonomic differences of the rumen microbiome

**Fig 2:**
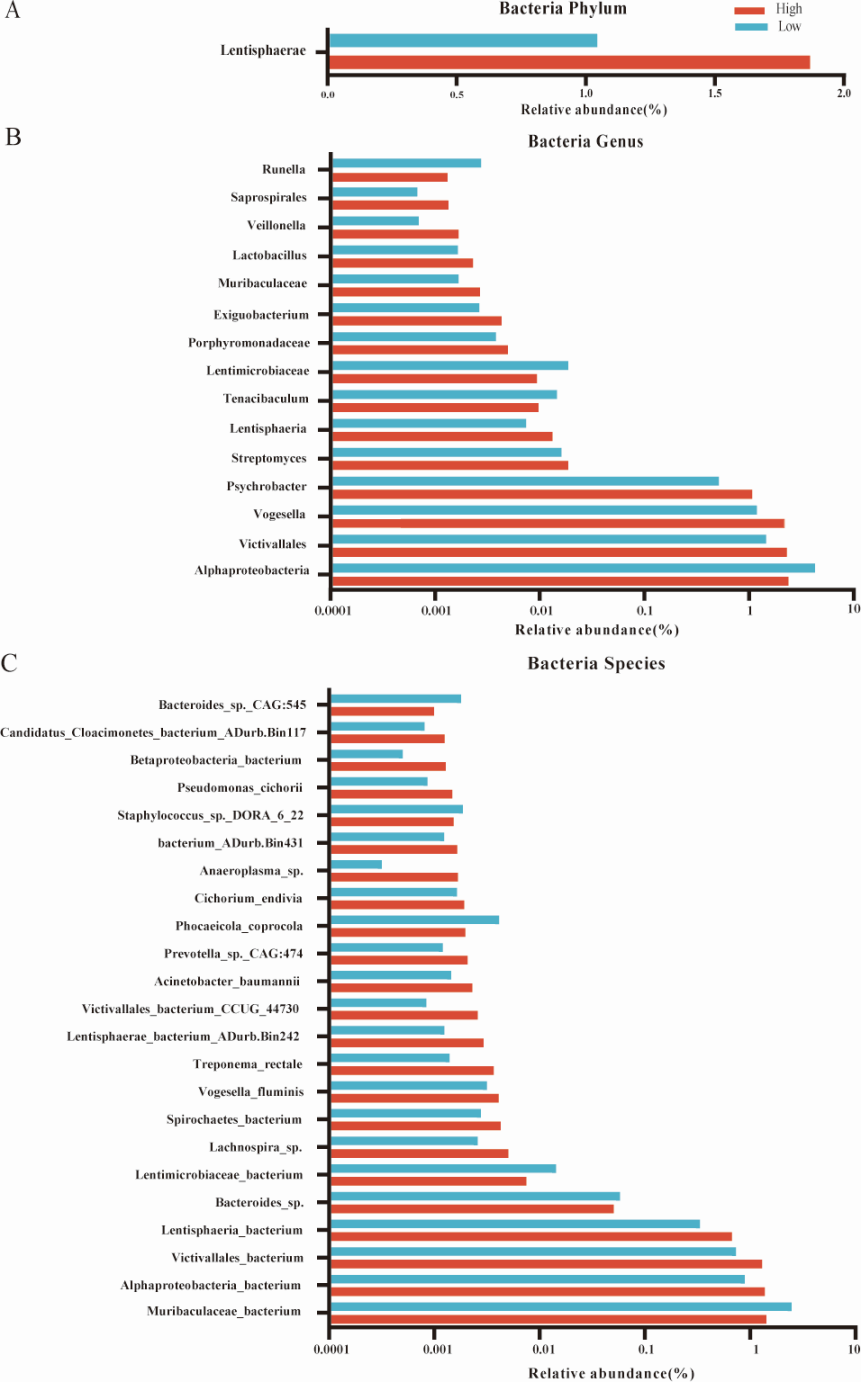
Differential rumen microbial species between the High group and Low group. Significantly different microbiomes on phylum level (**A**), genus level (**B**), and species level (**C**). Significant differences were tested by linear discriminant analysis effect size analysis, with relative abundance >0.001% and *P* value of <0.05.

The predominant bacterial phyla in the rumen microbiome were Bacteroidota and Firmicutes (Fig. S1A and B), while the most abundant genera included unclassified_o__Bacteroidales, followed by Prevotella, unclassified_f__Lachnospiraceae, and unclassified_f__Paludibacteraceae (Fig. 1D and E). The dominant species within the rumen were Bacteroidales bacterium, Prevotella sp., Paludibacteraceae bacterium, Clostridia bacterium, Lachnospiraceae bacterium, and Bacilli bacterium (Fig. 1F and G). Differential abundance analysis between the High and Low groups revealed notable taxonomic differences. At the phylum level, Lentisphaerae was significantly more abundant in the rumen of the High group (relative abundance >0.001%, *P* < 0.05; Fig. 2A). At the genus level, 10 genera, including Streptomyces, Lactobacillus, Vogesella, and Tenacibaculum, were significantly enriched in the High group, whereas five genera were significantly more abundant in the Low group (relative abundance >0.001%, *P* < 0.05; Fig. 2B). At the species level, 17 species exhibited significantly higher abundances in the High group, including two Lentisphaerae bacteria, two Victivallales bacteria, and one Alphaproteobacteria bacterium (relative abundance >0.001%, *P* < 0.05). Conversely, six species showed significant enrichment in the rumen of the Low group (relative abundance >0.001%, *P* < 0.05; Fig. 2C).

### Functional profiles of the rumen microbiome and differential functions between the two groups

**Fig 3:**
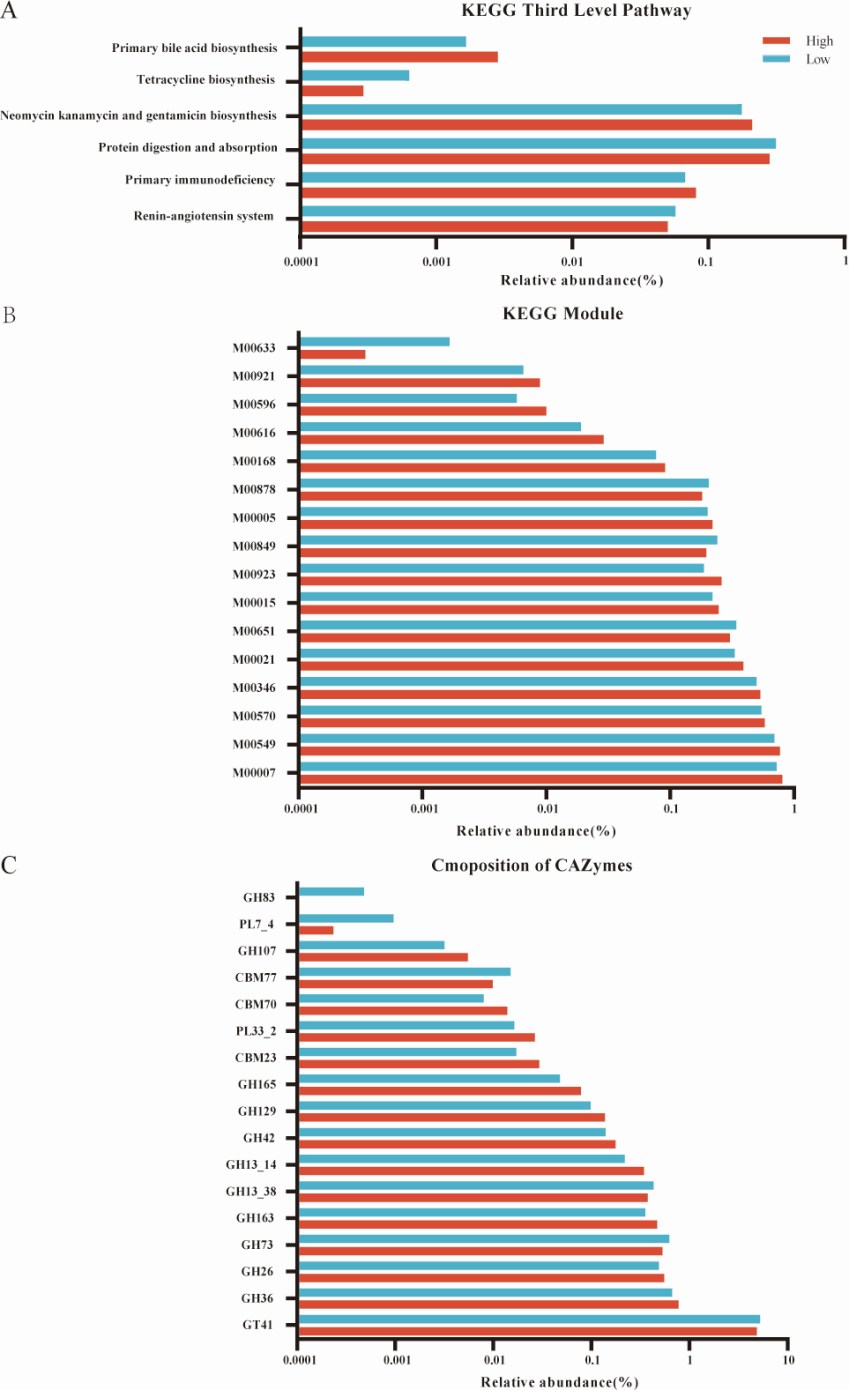
Differential KEGG functions and carbohydrate-active enzymes (CAZymes) analysis between the High group and Low group. Significantly different third-level pathway of KEGG functions (**A**). Comparison of rumen microbial KEGG modules (**B**) and CAZymes (**C**) between the two groups.

A total of 443 endogenous pathways at the third level were identified, representing the metabolic functions of rumen microorganisms. These pathways were classified into six broad categories: “Metabolism” (68.18%), “Genetic Information Processing” (8.60%), “Environmental Information Processing” (5.44%), “Cellular Processes” (5.17%), “Organismal Systems” (5.79%), and “Human Diseases” (6.85%). At the second level, 47 distinct pathways were identified, with the most abundant including “Carbohydrate metabolism” (9.19%), “Amino acid metabolism” (4.87%), “Replication and repair” (3.99%), and “Glycan biosynthesis and metabolism” (3.88%) (Table S9). Upon comparison of the identified Kyoko Encyclopedia of Genes and Genomes (KEGG) pathways, six third-level pathways showed significant differences, including three related to “Metabolism” (ko00524, ko00120, and ko00253), two related to “Organismal Systems” (ko04973 and ko04614), and one related to “Human Diseases” (ko05340) (Fig. 3A, *P* < 0.05). Further comparison of KEGG modules associated with these differential pathways revealed 14 modules enriched in the High group and eight in the Low group (Fig. 3B, *P* < 0.05). Specifically, in the context of carbohydrate and amino acid metabolism, five modules were enriched in the rumen of High group Xuzhou cattle (M00007, M00057, M00021, M00015, and M00005), while only one module (M00007) was enriched in the Low group.

In total, 565 genes encoding carbohydrate-active enzymes (CAZymes) were identified (Table S10). These included 21 auxiliary activities, 71 carbohydrate-binding modules (CBMs), 15 carbohydrate esterases (CEs), 272 glycoside hydrolases (GHs), 100 glycosyltransferases (GTs), 85 polysaccharide lyases (PLs), and one Cellulosome Module (surface layer homology). Among these, genes encoding GT2_Glycos_transf_2 (7.77%) were the most abundant, followed by GH2 (5.74%), GT4 (5.00%), and GT41 (4.08%). Of the top 20 carbohydrate enzymes by relative abundance, four glycosyltransferases were most prominent (GT2_Glycos_transf_2, GT4, GT41, and GT83) and 11 glycoside hydrolases, including GH2, GH97, GH3, GH20, GH92, GH78, GH31, GH29, GH28, GH106, and GH95. Additionally, the most abundant carbohydrate esterases were CE1, CE10, and CE4.

A comparison of CAZymes involved in the degradation of carbohydrates, such as cellulose, hemicellulose, starch, protein, and lignin, revealed that 10 CAZymes (8 GHs and 2 PLs) were enriched in the rumen of the High group, while three (all GHs) were enriched in the Low group. Among the GTs responsible for carbohydrate synthesis, only one (GT41) was enriched in the Low group. Notably, two carbohydrate-active enzymes involved in the degradation of complex carbohydrates, which are non-catalytic in nature, were enriched in the rumen of the High group, while only one was enriched in the Low group (Fig. 3C, *P* < 0.05).

### Interactions between rumen microbiome and rumen metabolites associated with the host’s average daily gain

**Fig 4:**
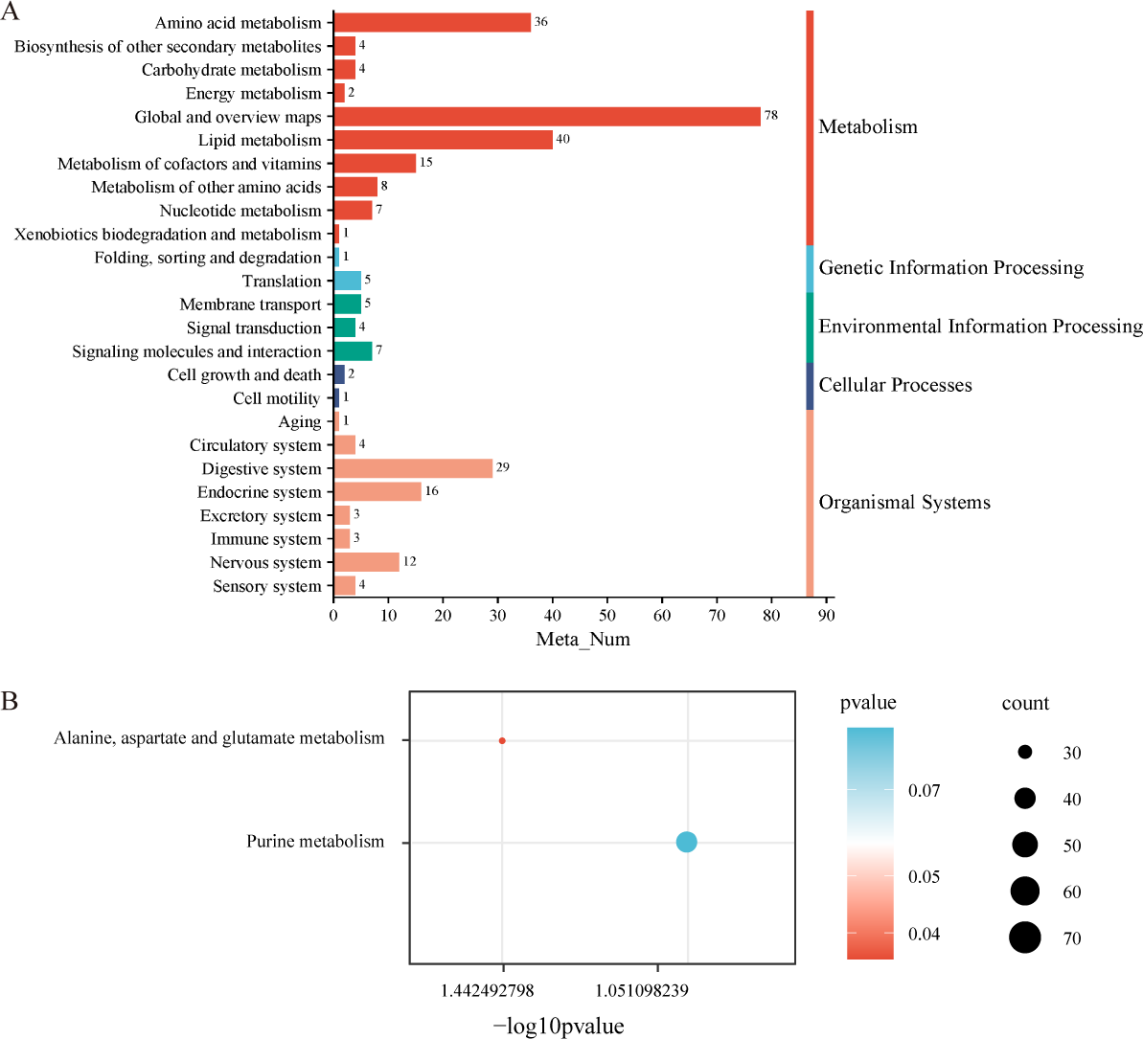
Rumen metabolome of the High group and Low group. (**A**) Pathway enrichment analysis was performed using the rumen metabolites of the High group and the Low group in total. (**B**) Pathway enrichment analysis was performed based on the significantly different rumen metabolites between the High group and the Low group. Significant differences were tested by linear discriminant analysis effect size analysis, with relative abundance >0.001% and *P* value of <0.05.

To further elucidate the relationship between the rumen metabolome, microbiome, and host phenotypes, we conducted rumen fluid metabolome sequencing and identified a total of 577 metabolites (Table S11). Of these, 252 metabolites were detected in the negative ion (neg) state, while 325 metabolites were observed in the positive ion (pos) state. Based on these metabolites, 25 second-level metabolic pathways were enriched (Fig. 4A), with the majority belonging to the “Metabolism” category. Ten of the second-level pathways were related to various metabolic processes. A *t*-test combined with variable importance in projection (VIP) filtering revealed 10 metabolites exhibiting significant differences between the High group and Low group. Among these, three metabolites were up-regulated, and seven were down-regulated (Table 1).

**TABLE 1:** Differential metabolites between the two groups

Name	log_2_FC	*P* value	Up_Down
Gamma-glutamyltyrosine	−0.6	<0.01	Down
D and C Yellow 11	−0.8	<0.01	Down
Asp-Phe	−0.8	<0.05	Down
Gly-Phe	−0.6	<0.05	Down
Loxoprofen	−1	<0.05	Down
N-acetylaspartic acid	−0.8	<0.05	Down
2′-Deoxyinosine	−1.4	<0.05	Down
LPE 12:0	1.75	<0.05	Up
2-Oxa-4-azatetracyclo[6.3.1.1~6,10~.0~1,5~]tridecan-3-one	1.39	<0.05	Up
(5Z)-3-aminonon-5-enoic acid	1.43	<0.05	Up

Metabolic pathway analysis was further performed on the 10 significantly different metabolites, and two third-level pathways were identified as being enriched, specifically ko00230 and ko00250. Notably, the “Alanine, aspartate, and glutamate metabolism” pathway (ko00250) was significantly different between the two groups (*P <* 0.05, Fig. 4B; Fig. S2C).

**Fig 5:**
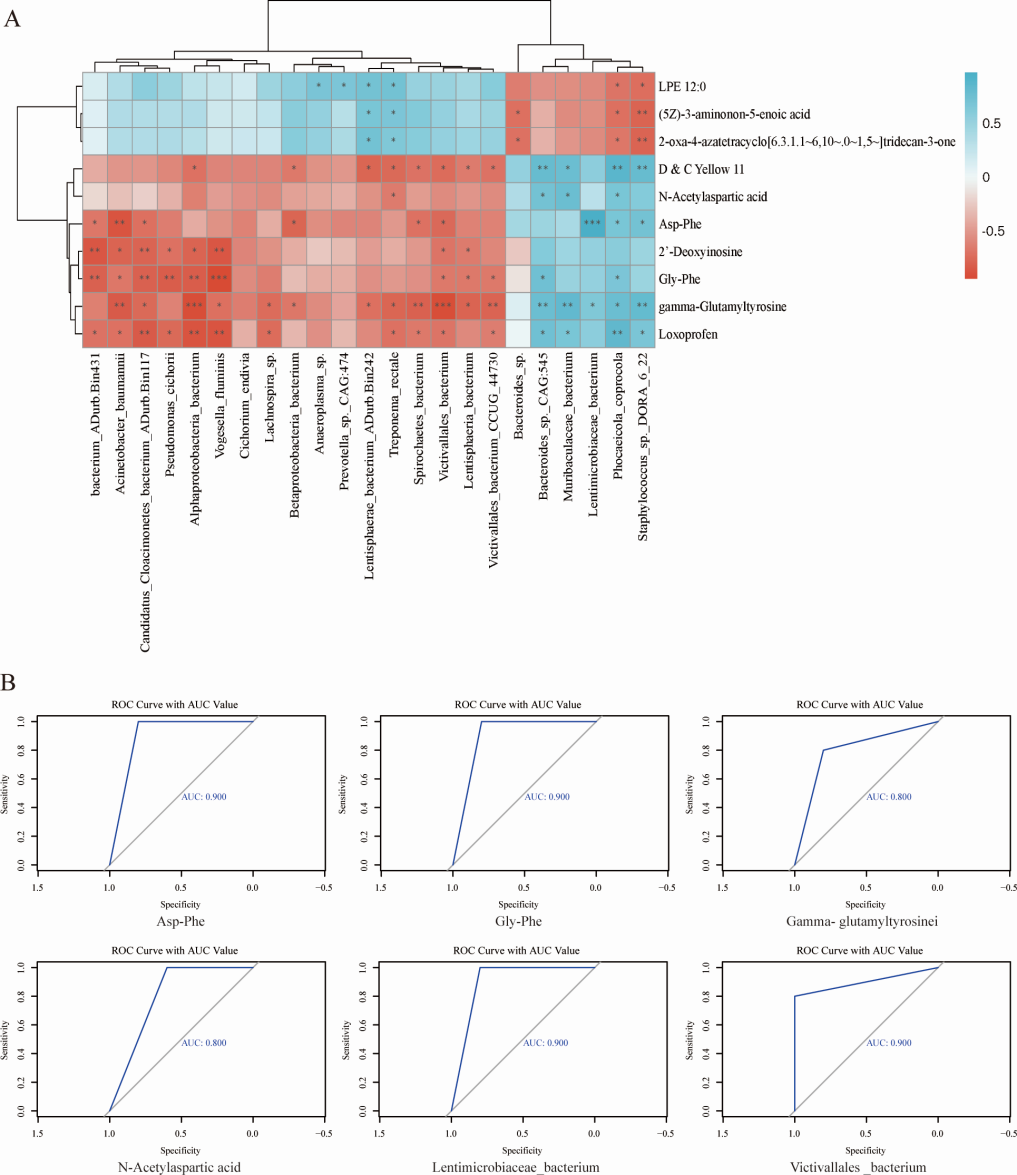
Interactions between rumen microbiomes and metabolites. Prediction of the effect of average daily gain using rumen metabolites and microbe-metabolite interactions. Receiver operating characteristic (ROC) curve and the confusion matrix of the performance of the random forest model using the four selected metabolites and two microbiomes, whose means are the highest. (**A**) Spearman’s correlations between rumen microbiomes and rumen microbial metabolites. (**B**) The ROC curve with area under the curve (AUC) value of four metabolites and two microbiomes. Only strong correlations (*R* > 0.05 or *R* < −0.5, *P* < 0.05) were shown in the correlation networks.

Spearman’s rank correlation analysis between the rumen microbiome and metabolites revealed 92 significant correlations (*R* > 0.50, *P <* 0.05; Fig. 5A). Among these correlations, five key rumen microbiomes were positively correlated with amino acids, peptides, proteins, and organic chemicals. These include Phocaeicola coprocola, Lentimicrobiaceae bacterium, Muribaculaceae bacterium, Lentisphaerae bacterium ADurb.Bin242, and Treponema rectale.

Based on these correlations, 23 rumen microbiomes and 10 rumen metabolites were selected for association with host ADG. These were subsequently used to construct a random forest model for prediction, and the receiver operating characteristic (ROC) curve was plotted based on the IncNodePurity index. The results demonstrated that the accuracy values of two keystone bacteria (Victivallales bacterium and Lentisphaerae bacterium) and four significant metabolites (gamma-glutamyltyrosine, Asp-Phe, N-acetylaspartic acid, and Gly-Phe), whose means are the highest (Tables S12 and S13), and Asp-Phe were the highest, indicating their strong association with ADG (Fig. 5B).

## DISCUSSION

In this study, the Xuzhou cattle were divided into the High group and Low group, and the rumen microbiomes of the two groups of Xuzhou cattle were compared and analyzed, and their functions were predicted (32, 33). The sequencing results of the rumen metagenome revealed that the dominant bacterial phyla were Bacteroidota and Firmicutes in the Xuzhou cattle, consistent with previous studies by Liu et al. (34), Zhao et al. (35), Zhou et al. (36), Ames et al. (37), and Han et al. (38). Rumen microbiome had an important impact on fermentation and degradation of the feed. Some relevant studies have revealed that Bacteroidota and Firmicutes in the rumen could degrade non-starch polysaccharides of the plants (39). Moreover, Bacteroidota produce acetic acids (40), while Firmicutes can produce butyric acids (41) and play an important role in the degradation of crude fibers and heterozygous polysaccharides. The dominant bacterial genus was unclassified_o__Bacteroidales and Prevotella. A large number of studies have revealed that Bacteroides was recognized as one of the main genera in the core microbial community module. Bacteroidota have a close symbiotic relationship with the host, which can stimulate the immune system of the body (42), enhance phagocytosis, induce the proliferation of probiotics, and improve the immunity of animals. Prevotella belonged to Prevotellaceae, which is part of the Bacteroidota. This genus could produce acetate and succinic acid by using starch and protein. Similar results were observed in other ruminants. For example, Li et al. (43) reported the relative abundance of Prevotella in the rumen and large intestine was the highest in terms of digestibility and rumen fermentation of Hu sheep, which was consistent with the research results of Brulc et al. (44) and Pitta et al. (45). In addition, some studies revealed that Prevotella has the ability to degrade mucinous glycoprotein (46), which can not only promote the growth and increase the survival rate of the host, but also degrade hemicellulose and xylan, thus promoting the digestion of feed. At the species level, Bacteroides and Prevotella (both Bacteroidota) dominated the rumen microbiota of Xuzhou cattle.

Notably, differential abundance analysis at the phylum level showed that Lentisphaerae was significantly enriched in the High group. According to relevant reports, Lentisphaera was widely distributed in seawater (47), anaerobic sludge (48), landfill leachate (49), sediment (50), animal and human gastrointestinal tract (51, 52). In 2004, scientists isolated and cultivated two distinct marine strains from seawater collected along the Oregon coast, labeling them HTCC2155(T) and HTCC2160. They proposed classifying these strains within a novel genus and species, termed Lentisphaera araneosa (53), with HTCC2155(T) serving as the type strain [equivalent to ATCC BAA-859(T) and KCTC 12141(T)]. This proposal introduced a new genus (gen. nov.) and a new species (sp. nov.) to the scientific community. Most of the studies on Lentisphaera are based on metagenomic predictions. For example, Das et al. (54) found that Lentisphaera had a bioremediation function; Yan et al. (55) revealed that Lentisphaera had the function of degrading polysaccharides. In addition, Lentisphaera has been closely associated with human health problems (56). In the analysis of metabolite correlation of rumen microbiomes, Lentisphaera was significantly negatively correlated with gamma-glutamyltyrosine, Gly-Phe, and 2′-deoxyinosine.

The relative abundance of 11 bacteria at the genus level was significantly identified in the High group, such as Streptomyces, Lactobacillus, Vogesella, and Tenacibaculum. The secondary metabolite of Streptomyces has strong antibacterial activity, and about 75% of the industrial antibiotics are derived from streptomycin at present (57). One of the scientists and his team from the Institute of Software, Chinese Academy of Science, called Lin Hongyu, isolated the secondary metabolite of Streptomyces called Lu01-M, which could inhibit the proliferative ability of prostate cancer cells (58). Lactobacillus, belonging to common lactobacillus, is a probiotic. Relevant studies revealed that Lactobacillus was highly related to the maintenance of microbial homeostasis, animal immunity, body health, and the improvement of animal growth rate (59, 60). Moreover, scientists (61) from the Department of Microbiology and Alimentary Pharmabiotic Centre, University College Cork, Cork, Ireland, revealed that Lactobacillus plantarum had the ability to inhibit the growth of harmful bacteria such as *Escherichia coli* and could resist the invasion of pathogenic microbiomes to ensure the healthy growth of animals. In addition, Lactobacillus has the ability to ferment multi-carbohydrate feeds and improve pork quality (62). The relative abundance of four bacteria at the genus level was higher in the Low group, including Muribaculaceae, Porphyromonadaceae, Lentimicrobiaceae, and Psychrobacter. Relevant studies have shown that Psychrobacter, Salegentibacter, and Jeotgalicoccus have low temperature tolerance (63), high salt tolerance (64), and thermal stability (65), respectively, and these microbial functions may be related to the adaptability of Xuzhou cattle. Differential abundance comparison analysis between two groups at the species level showed that 17 bacteria were significantly identified in the High group, including Alphaproteobacteria_bacterium, Victivallales_bacterium, Lentisphaerae_bacterium, and Victivallales_bacterium, Lentisphaerae_bacterium, Lentimicrobiaceae_bacterium, Lentisphaerae_bacterium_ADurb.Bin242, and Victivallales_bacterium_CCUG_44730 all belonged to Lentisphaera.

As numerous studies have reported, the functional characteristics of the rumen microbiome exhibit greater conservation than the taxonomic composition when compared across different groups of animals. Through KEGG function analysis, it was found that carbohydrate metabolism and amino acid metabolism were the most abundant functions, which was consistent with the result of Khatoon et al. (66). When the identified KEGG pathways were compared, KEGG functions on carbohydrate metabolism and amino acid metabolism were enriched in the rumen of the High group. For example, the conversion of fructose 6-phosphate to ribose 5-phosphate (M00007), threonine to 2-oxo-butyric acid, and then to isoleucine (M00057) and serine to cysteine (M00021) are other modules, indicating that carbohydrate metabolism and amino acid metabolism are more active in the High group. These results imply that the High group microbiota may exhibit enhanced carbohydrate degradation capacity and may produce more hydrolysates and pyruvate.

CAZymes are a very important class of enzymes, divided into six categories, including glycoside hydrolases, glycosyltransferases, polysaccharide lyases, and glycoesterases, which have the functions of degrading, modifying, and generating glycoside bonds. Further study on CAZyme is of great significance to reveal the metabolic mechanism of microbial carbohydrates. According to the number of genes in the CAZy database annotated, GH enzymes were the most abundant statistically, which was consistent with the study results of Kala et al. (67) and Torres Manno et al. (68). When the identified CAZymes were compared, nine GHs, one CBM enzyme, and one PL were found among the 11 enzymes significantly enriched in the High group, and relevant studies have shown that GH enzyme has the ability to hydrolyze cellulose, and CBM enzyme participates in enzyme-substrate binding (69).

Based on the enrichment analysis of the metabolic pathways of the above 10 different metabolites, two metabolic pathways were obtained, namely Alanine, aspartate, and glutamate metabolism, and Purine metabolism. Alanine, aspartate, and glutamate metabolism were significantly different between the two groups. In relevant studies, goats were used as animal models to study the relationship between rumen microbial composition and rumen metabolome (70). In this study, the correlation between rumen metagenome and rumen metabolism was determined through correlation analysis of rumen microorganisms and metabolites. We found that microorganisms with significant differences in the High group were negatively correlated with amino acid metabolites. For example, Victivallales_bacterium is negatively and significantly correlated with gamma-glutamyltyrosine, Asp-Phe, N-acetylaspartic acid, and Gly-Phe. In order to further explore the relationship between rumen microbial metabolites and individual daily gain of Xuzhou cattle, the random forest model was used to predict, and statistical analysis found that the two metabolites with the highest IncNodePurity index were Asp-Phe (area under the curve [AUC] = 0.900) and Gly-Phe (AUC = 0.900). The two microorganisms are Muribaculaceae_bacterium (AUC = 0.700) and Victivallales_bacterium (AUC = 0.700). This result aligns with metagenomic and metabolomic analyses, suggesting a robust link between microbial composition and metabolic activity.

Although we greatly controlled for factors affecting ADG during the experiment, such as animal diet, animal age, animal sex, and feeding management, we also found that the daily gain of the host may also be related to changes in rumen microbial composition and metabolites, or host digestion and absorption of metabolites. In addition, the host’s daily gain may also have a strong relationship with animal feed intake, as well as genetic aspects. Relevant studies have shown that heredity can affect not only animal phenotype but also microbial composition (71). At present, there are few reports about the function of microorganisms and their metabolites, and the mechanism of microbial influence on meat production traits. For example, the causal relationship among feed intake, microbial community dynamics, and ADG can be analyzed by designing gradient feed intake experiments, combined with continuous sampling and microbiome timing analysis in the future. Therefore, further research on the function of microorganisms and their heritability is needed in the future. In addition, this study lacks validation tests and needs further proof.

### Conclusion

There were 10 metabolites in the rumen of Xuzhou cattle with significant differences. Based on the metabolic pathway analysis of these 10 metabolites, the two metabolic pathways obtained were related to amino acid metabolism. Correlation analysis showed that Victivallales_bacterium was negatively correlated with gamma-glutamyltyrosine, Asp-Phe, N-acetylaspartic acid, and Gly-Phe. To sum up, two kinds of microorganisms (Muribaculaceae_bacterium and Victivallales_bacterium) and four kinds of metabolites (gamma-glutamyltyrosine, Asp-Phe, N-acetylaspartic acid, Gly-Phe) interaction had a positive effect on the daily gain phenotype of Xuzhou cattle. This study elaborated the regulatory mechanism of rumen microbial metabolites on host daily gain phenotype, which provided a theoretical basis for the feeding management and nutritional regulation of beef cattle.

## MATERIALS AND METHODS

### Experimental animals and feeding management

**Fig 6:**
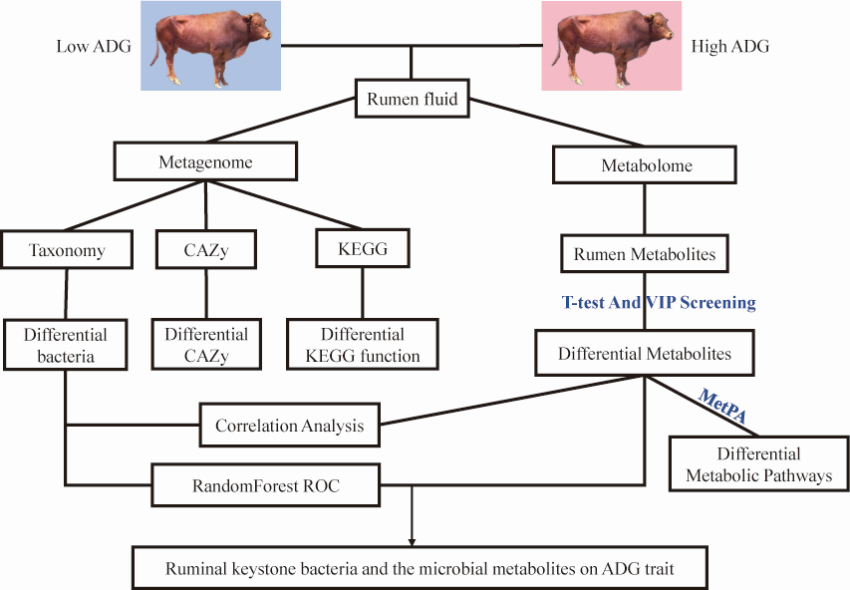
Workflow of the integrated rumen metagenomes and metabolomes on average daily gain in Xuzhou cattle.

In this study, Xuzhou cattle that were healthy, had similar body weight, received unified epidemic prevention and immunization, unified feeding and management, were fed regularly and in fixed quantities (with a ratio of concentrate to roughage of 3:7, and the roughage was corn silage and dry wheat straw), had free drinking water, and had no history of gastrointestinal diseases or metabolism were selected as the experimental cattle herds. During the breeding period, the exercise volume of the cattle herd should be uniformly controlled, and disease prevention and control should be carried out. The experimental design is presented in Fig. 6. According to the feeding standard of the fattening cattle in NY/T 815, the cattle were raised to 24 months of age, and the feeding experiment lasted for 180 days. After the experiment, 10 extreme individuals were selected from the herd according to the weight difference of the first and last measurements, and they were divided into High daily gain recombination (High group) and Low daily gain recombination (Low group). The daily weight gain interval of the High group was 0.66–0.74 kg, and the average daily gain was 0.72 ± 0.03 kg. The weight gain range of the Low group was 0.49–0.62 kg, and the average daily gain was 0.57 ± 0.05 kg (Table S14).

### DNA extraction, metagenomic sequencing, functional annotation, and data processing of rumen microbiome

The DNA of the rumen microbiome was extracted from two groups of 10 Xuzhou cattle according to the instructions of the Fast DNA SPIN soil kit (MP Biomedicals, Santa Ana, CA, USA). The concentration and purity were determined by ultraviolet spectrophotometer, and the DNA integrity was detected by 1% agarose gel electrophoresis. Covaris M220 instrument was used to fragment DNA, the length of which was about 350 bp, and a PE library was constructed. The amplified products were purified and sequenced by the Illumina HiSeq Xten platform. The raw metagenomic sequencing reads were first subjected to rigorous quality control using Fastp (v0.20.0). Initially, low-quality bases with Phred Q-scores <20 (corresponding to an error rate ≤1%) were trimmed from both ends of the reads. Subsequently, entire reads were discarded if they contained >10% ambiguous bases (N bases) or if their length after trimming fell below 50 bp. Following these initial filtering steps, adapter sequences were identified and trimmed, with only reads showing ≤5% residual adapter content being retained for downstream analysis. Finally, to ensure high overall read quality, we required that at least 80% of bases in each read maintain a Q-score ≥30, which represents the standard quality threshold for Illumina HiSeq X Ten platform data. Then, optimized reads were optimized for removal of host genes using BWA (72) (v07.17). The reads with the host DNA sequences removed were assembled by using MEGAHIT (73) (v1.1.2), and the OFR prediction was conducted based on the sequences with good assembly results (74). The obtained gene sequences were processed using the CD-HIT (75) software (v4.6.1) to identify non-redundant contigs. The longest gene in each category was selected as the representative sequence for constructing a non-redundant gene set.

Using DIAMOND software (76) (v2.0.13), we compared the non-redundant gene set with the NR database (comparison type: BLASTP) and obtained species annotations through the taxonomic information database corresponding to the NR library. Then we calculated the abundance of the species using the sum of the corresponding gene abundance. The abundance of species in each sample was counted at the taxonomic levels of Domain, Kingdom, Phylum, Class, Order, Family, Genus, and species. An abundance profile was constructed at the corresponding taxonomic level. This experiment utilized the Wilcoxon signed-rank test to compare the differential abundance and considered the false discovery rate-adjusted q-value <0.05 as significant.

KEGG database is a systematic analysis of gene function, contact genome, and function of a large knowledge base of information. In this study, the predicted gene protein sequences were compared with the KEGG database based on BLAST comparison, and the gene function annotation results were obtained.

Carbohydrates-active enzyme (CAZy) is about the synthesis of active databases or complex carbohydrates and sugar compound enzymes professional database. The corresponding tool hmmscan of the CAZy database was used to compare the non-redundant gene set with the CAZy database (v5.0), and the expected value of the comparison parameter set was 1e-5 (77), so as to obtain annotation information of carbohydrate active enzymes corresponding to the gene. Then the abundances of carbohydrate-active enzymes were calculated using the sum of gene abundances corresponding to carbohydrate-active enzymes.

### Rumen metabolism group sample processing and data processing

Take 100 µL of rumen fluid sample, add 400 µL of 80% methanol aqueous solution, vortex-mix for 1 min, let the sample stand at −20°C for 30 min, incubate after that, place the mixture at 4°C, centrifuge at 12,000 rpm for 20 min, take 150 µL of the supernatant. Blow-dry with nitrogen, resuspend the supernatant with 150 µL of 80% methanol solution containing 2-chloropropylalanine, and filter through a 0.22 µm sterile membrane to obtain the test sample. To ensure the consistency of processing and detection, mix 50 µL of each sample to make QC samples. Analyze by liquid chromatography-mass spectrometry (LC-MS), design five samples together with QC samples every five samples to evaluate the stability of detection during the analysis process. During the LC-MS analysis process, the system stability was first monitored through periodically inserted QC samples (one QC for every five analysis samples). The retention time (RSD) of the internal standard metabolites in the QC samples was always less than 2%, indicating stable chromatographic separation. Meanwhile, the RSD of the peak area of characteristic metabolites was controlled within 15% (20% for lipids). And through real-time monitoring of quality accuracy (Orbitrap <5 ppm) and baseline signal-to-noise ratio (S/*N* ≥ 50).

The processed samples were analyzed by gas chromatography combined with Pegasus HT time-of-flight mass spectrometry (78). The positive and negative ion modes were collected using a high-resolution tandem mass spectrometer, Xevo G2-XS QTOF. The peaks, extracted peaks, normalization, deconvolution, and compound identification were performed using Progenesis QI (v2.2) software, and the extracted data were filled and filtered using the R “metaX” package (79). The possible molecular formulas of metabolites were determined, and data with mass errors >−10 ppm were excluded. Further screening was conducted.

The data files were processed using Compound Discoverer 26 software (80) (v3.1), and retention time and mass-to-charge ratio screening were performed for each metabolite. Peak alignment was conducted between different samples with a retention time deviation of 0.2 min and a mass deviation of 5 ppm to improve identification accuracy. During peak extraction, a mass deviation of 5 ppm, a signal intensity deviation of 30%, and a signal-to-noise ratio of 3 were used. Based on the molecular ion peaks and fragment ion peaks, the target ions were integrated, and the molecular formulas were predicted. Comparisons were made with the mzCloud, mzVault, and Masslist databases. Background ions were removed using blank samples, and the original quantitative results were standardized using the formula of the sample’s original quantitative value/(total quantitative value of sample metabolites/QC sample total quantitative value) to obtain relative peak areas. After removing compounds with a relative peak area coefficient of variation of relative peak areas >30% from all QC samples, the metabolite identification and relative quantitative results were finally obtained.

Orthogonal partial least squares discriminant analysis (OPLS-DA) was performed using the R package “cord” (v1.6.2), and sevenfold cross-validation was conducted to evaluate the stability of the model. Significant differential metabolites were selected based on Student’s *t*-test and the multivariate analysis of the OPLS-DA model, with VIP-values >1 and *P* values <0.05. Metabolic pathway annotation was performed using the KEGG database based on differential metabolites.

### Data processing

Alpha diversity analysis was often used to reflect the number, abundance, and distribution of species in a sample. In this study, Sample, Chao, Shannon, Simpson, Coverage, and Pielou_e index were selected as parameters for Alpha diversity analysis, and QIIme (v1.9.1) was used to calculate the above indices. Visualization was performed using R software (v4.4.0) to reflect the microbial community diversity between the High group and the Low group, and the difference analysis of the Alpha diversity index was performed by using a *t*-test.

PCoA was used to study the method of similarities or differences and visual data. PCoA was commonly used to process the matrix of distances between genotype data, samples, or species abundance. The PCoA was drawn using R software (v4.4.0). Wilcoxon rank and *t*-test were used to compare the composition of microbiome phylum, genus, and species levels between the two groups, and significant differences were considered by *P* value <0.05. LEfSe was used to compare ruminal bacteria species, R software (v4.4.0) was used for statistics and mapping based on the relative abundance table of genes, species, and functions, and R software (v4.4.0) “vegan” package was used for data clustering and abundance analysis, and bacteria diversity, composition differences, and functional differences were analyzed (including KEGG and CAZy). In addition, differential metabolite screening and functional analysis were performed using R software (v4.4.0) “ropls” package (81). The online platform called MetaboAnalyst 4.0 (Meta) (82) was used to enrich the metabolic pathway (83). Based on the enrichment data of metabolite metabolic pathways, visual analysis was performed using the “ggplot2” package in R software (v4.4.0).

Comprehensive correlation analysis was conducted using the “Spearman rank” correlation method, a non-parametric statistical approach particularly suited for detecting monotonic relationships in microbial ecological data. The resulting correlation matrix was visualized as a “heatmap,” constructed using the “pheatmap” package in R software (version 4.3.1), which enabled the hierarchical clustering of both bacterial taxa and metabolites based on their correlation patterns. This visualization not only highlighted significant positive and negative associations but also revealed potential functional modules within the rumen ecosystem.

To further identify the most predictive microbial and metabolic biomarkers, a machine learning-based Random Forest analysis was performed using the “RandomForest” package in R. The importance of rumen microbiome and metabolite characteristics was ranked according to their contribution to the accuracy of model prediction. The optimal feature set was determined by maximizing the “AUC” of the ROC curve, a metric that quantifies the model’s ability to discriminate between classes. The “UC-RF algorithm” (Unbiased Conditional Random Forest) was specifically utilized to mitigate potential biases in variable selection, ensuring the reliability of the identified biomarkers.

## Data Availability

Raw data are available at Bioproject: PRJNA1227409.

## SUPPLEMENTAL MATERIAL

Supplemental figures spectrum.00769-25-s0001.pdf Fig. S1 and S2.

Table S1 spectrum.00769-25-s0002.xlsx Sequencing results.

Supplemental tables spectrum.00769-25-s0003.xlsx Tables S2 to S8.

Table S9 spectrum.00769-25-s0004.xlsx Composition of metabolic pathways based on the first-level and second-level functions in the KEGG.

Table S10 spectrum.00769-25-s0005.xlsx Composition of CAZymes.

Table S11 spectrum.00769-25-s0006.xlsx Results of rumen metabolome analysis.

Table S12 spectrum.00769-25-s0007.xlsx The IncNodePurity of rumen microbiomes.

Table S13 spectrum.00769-25-s0008.xlsx The IncNodePurity of rumen microbiomes.

Table S14 spectrum.00769-25-s0009.xlsx Results of daily weight gain of Xuzhou cattle population.

ASM does not own the copyrights to Supplemental Material that may be linked to, or accessed through, an article. The authors have granted ASM a non-exclusive, world-wide license to publish the Supplemental Material files. Please contact the corresponding author directly for reuse.
