# Supplementary material for: Integrated multi-omics reveals the impact of ruminal keystone bacteria and microbial metabolites on average daily gain in Xuzhou cattle
Source: Microbiol Spectr. 2025 Jun 30;13(8):e00769-25. doi: 10.1128/spectrum.00769-25 (PMC12323375; doi:10.1128/spectrum.00769-25)
Supplement: Supplemental figures — Fig. S1 and S2. [file spectrum.00769-25-s0001.pdf]

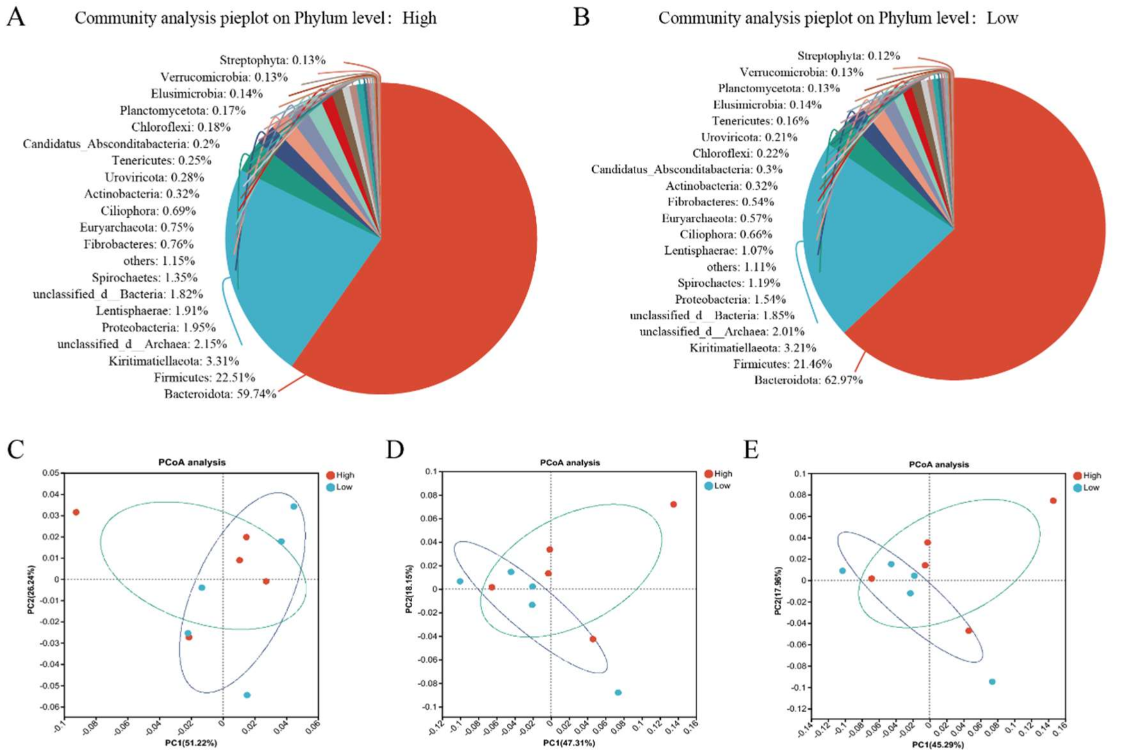

Figure. S1 Microbial compositional profiles of two groups. Community analysis pieplot of High Group **(A)** and Low Group **(B)** on phylum level. High Group and Low Group based on species visualized using principal-coordinate analysis (PCoA) of Xuzhou cattle on phylum level **(C)**, genus level **(D)** and species level **(E)**

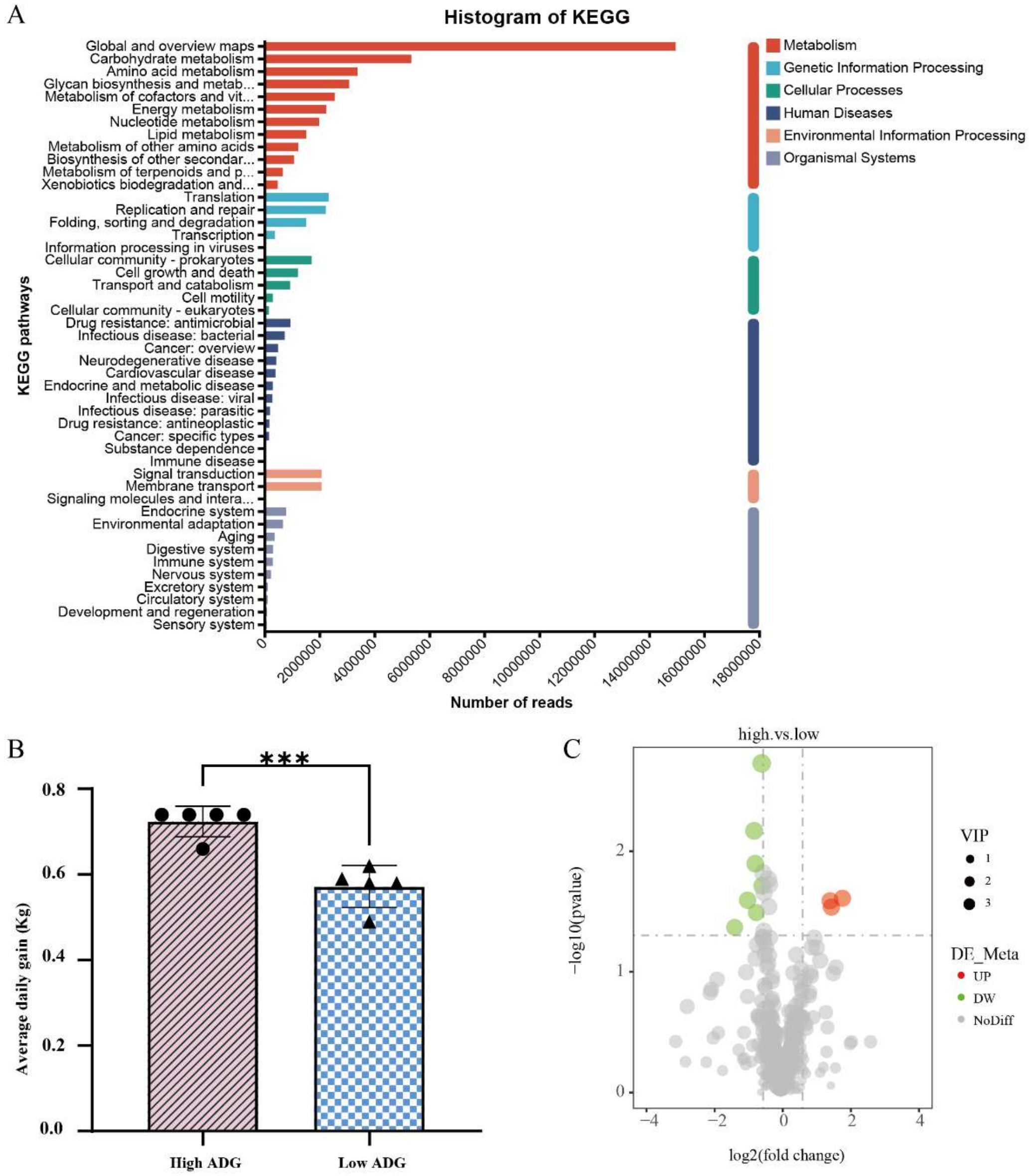

Figure. S2 Grouping results, microbial KEGG functions at the level 3 pathway, and volcanic maps of significantly different metabolites of Xuzhou cattle. **(A)** Pathway enrichment analysis performed using the microbial KEGG functions of two groups. (**B)** Grouping results of High and Low. (C) Volcanic maps of significantly different metabolites.
